# Characterizing Facial Skin Ageing in Humans: Disentangling Extrinsic from Intrinsic Biological Phenomena

**DOI:** 10.1155/2015/318586

**Published:** 2015-02-12

**Authors:** Carina Trojahn, Gabor Dobos, Andrea Lichterfeld, Ulrike Blume-Peytavi, Jan Kottner

**Affiliations:** Clinical Research Center for Hair and Skin Science, Department of Dermatology and Allergy, Charité-Universitätsmedizin Berlin, Charitéplatz 1, 10117 Berlin, Germany

## Abstract

Facial skin ageing is caused by intrinsic and extrinsic mechanisms. Intrinsic ageing is highly related to chronological age. Age related skin changes can be measured using clinical and biophysical methods. The aim of this study was to evaluate whether and how clinical characteristics and biophysical parameters are associated with each other with and without adjustment for chronological age. Twenty-four female subjects of three age groups were enrolled. Clinical assessments (global facial skin ageing, wrinkling, and sagging), and biophysical measurements (roughness, colour, skin elasticity, and barrier function) were conducted at both upper cheeks. Pearson's correlations and linear regression models adjusted for age were calculated. Most of the measured parameters were correlated with chronological age (e.g., association with wrinkle score, *r* = 0.901) and with each other (e.g., residual skin deformation and wrinkle score, *r* = 0.606). After statistical adjustment for age, only few associations remained (e.g., mean roughness (*R*_*z*_) and luminance (*L* ^*^),  *β* = −0.507, *R* ^2^ = 0.377). Chronological age as surrogate marker for intrinsic ageing has the most important influence on most facial skin ageing signs. Changes in skin elasticity, wrinkling, sagging, and yellowness seem to be caused by additional extrinsic ageing.

## 1. Introduction

The human skin is a large and highly complex organ, consisting of different layers and cell types. It serves as a barrier between the external environment and the inside of the body. The skin fulfils a large range of functions, including prevention of percutaneous water loss, temperature maintenance, sensory perception, and immune surveillance [1]. Moreover, skin health and appearance play crucial roles for self-esteem and social interactions [2].

Skin ageing can be formally conceptualized into intrinsic and extrinsic ageing. Intrinsic skin ageing represents the “normal” course of ageing of all tissues strongly associated with chronological age [3]. Among others intrinsic ageing results in increased skin surface roughness, fine lines and subepidermal atrophy [4, 5]. Extrinsic ageing, mainly caused by exposure to ultraviolet and infrared radiation, pollution, and cigarette smoking is superposed on intrinsic skin ageing. Accumulation of these insults results in formation of deeper wrinkles and pigmentary changes [6, 7].

Depending on the anatomical site the human skin may be affected by both: intrinsic and extrinsic ageing. This is especially true for the face, which is exposed to numerous environmental factors during the whole life course, comparable to dorsal hand skin [8]. Additionally, repeated facial expressions aggravate the formation of wrinkles [9]. The appearance of the facial skin is most important for the perceived age [10, 11]. In western societies females seem to be very concerned about their facial appearance and visible ageing phenomena indicated by increasing expenditures for cosmetic products and aesthetic procedures [12].

Despite extensive research over the past decades, there is no consensus upon a biological marker for human ageing [13]. The variable “chronological age” still seems to be one of the best predictors for intrinsic biological ageing. This also seems to be true for skin ageing. In the field of dermatologic research various parameters are established to quantify biological and biophysical skin characteristics. Functional parameters like skin surface pH, stratum corneum hydration (SCH) or transepidermal water loss (TEWL) are for instance applied as markers of the status and integrity of the skin barrier [14]. During ageing skin surface pH may increase, whereas TEWL decreases [15] and structural assessments revealed rougher human skin [16, 17] and decreased biological elasticity [18]. Age-dependent changes have also been demonstrated for skin colour [19]. In addition to these instrumental measures numerous clinical skin ageing scales are used in skin research and aesthetic dermatology [20–22]. Wrinkles, dyspigmentation, and sagging are most important items of these scores [23].

Although numerous studies apply these parameters to quantify facial skin ageing, an overall comprehensive picture, whether and how clinical characteristics and biophysical parameters are associated with each other is missing. Furthermore, because chronological age is the most obvious predictor for intrinsic facial skin ageing, we know little about the actual impact of external influences on facial skin biology irrespective of chronological age. Therefore, the purpose of this explorative study was to characterize facial skin ageing in females of three age groups and to identify possible associations between biophysical and clinical characteristics taking chronological age into account.

## 2. Methods

### 2.1. Study Design and Setting

This explorative study was conducted at the Clinical Research Center for Hair and Skin Science, Department of Dermatology and Allergy, Charité-Universitätsmedizin Berlin, Germany (52.3°N, 13.2°E). Between January and April 2014 skin ageing related clinical assessments and biophysical skin measurements were applied in female subjects of three age groups. This study was conducted according to the principles of the current version of the Declaration of Helsinki.

### 2.2. Participants

Twenty-four Caucasian women with healthy skin were enrolled. Main eligibility criteria were nonsmoking, no hormonal replacement therapy in the past two years, no extreme sun exposure two month prior study, no antiageing interventions including microsurgery, dermabrasion and retinoids in the past 10 years. The goal of recruitment was to include equal numbers of participants in each of three age groups (30–40 years, 50–60 years, and 70–80 years). Written informed consent was obtained from all participants before enrolment. Subjects were instructed not to wash their face and not to use any cosmetics at the face at least 12 hours prior to the measurements.

### 2.3. Lifetime Sun Exposure

Participants self-completed a questionnaire to measure their own lifetime sun exposure that was developed based on the literature on sunlight exposure assessment [24, 25]. Items included questions about the daily time spent in the sun during each decade of life. Lifetime sun exposure (hours) was calculated by adding the annual hours of sun exposure.

### 2.4. Clinical Assessments

The clinical assessments of skin ageing, wrinkling, and sagging were performed on standardized photographs taken from each subject's face from a front and 45° right and 45° left view. To enhance the visibility of wrinkles images were captured with a “parallel polarized” lightening mode (Visia-CR, Canfield Scientific, Fairfield, NJ, USA). After an initial training, all photographs were evaluated independently by three experienced investigators in a random order.

Three clinical aspects of skin ageing were quantified.* Global facial skin ageing* was assessed based on an instrument developed by Allerhand et al. [26] covering the following aspects: milia (cheek and forehead), pigmented spots (cheek and forehead), fine lines (cheek and forehead), lines on lips, wrinkles (cheek, under eyes and upper lip), furrows between eyebrows, nasolabial folds, crow's feet wrinkles, and facial tissue slackening. Sum scores between 14 and 42 were possible.* Facial wrinkling* was assessed using a modified visual analogue scale (100 mm) according to O'Hare et al. [27] and* sagging* was assessed using the 5-point midface volume loss scale by Lorenc et al. [28]. These tools were selected based on the evidence about their reliability and validity compared to many other available instruments [23].

### 2.5. Biophysical Measurements

All biophysical measurements were performed under strictly controlled conditions with a room temperature of 22 ± 2°C and a relative humidity of 50 ± 10% and after an acclimatization period of 30 minutes. The measurements were performed on the right and left upper cheeks. Statistical analysis was conducted with the average of triplicate measurements.

Skin barrier function was evaluated by measurement of the* TEWL* using the Tewameter TM 300 (Courage & Khazaka Electronic GmbH, Cologne, Germany),* SCH* using the Corneometer CM 825 (Courage & Khazaka Electronic GmbH, Cologne, Germany), and* skin surface pH* using the Skin-pH-Meter PH 905 (all probes were applied with the MPA system of Courage & Khazaka Electronic GmbH, Cologne, Germany). All measurements were conducted according to current guidelines [14, 29, 30].

The* skin colour* was measured with the Spectrophotometer CM-700d and corresponding software (Konica Minolta Sensing Inc., Japan), which was daily calibrated using the black and white plates supplied by the manufacturer. Illumination D65 was used for the measurements. The degree of luminance and yellowness in the skin tone was evaluated as *L* ^*^ and *b* ^*^ in the CIE *L* ^*^ *a* ^*^ *b* ^*^ colour space, respectively, similar to others [31].

The Visioscan VC 98 camera and the corresponding software SELS 2000 (both Courage & Khazaka Electronic GmbH, Cologne, Germany) were used to measure* skin microtopography*. We have used the DIN/ISO parameters *R*_max⁡_ and *R*_*z*_ because previous studies showed that these are the parameters with high reliability and validity estimates in elderly subjects [32]. While *R*_max⁡_ represents the maximal roughness, *R*_*z*_ is defined as the arithmetic mean roughness from five consecutive sampling lengths.

*Skin elasticity* was measured with the Cutometer MPA 580 and its accompanying software (Courage & Khazaka Electronic GmbH, Cologne, Germany). A probe opening of 2 mm diameter and a pressure of −450 mbar were used. Measurements were performed after three subsequent cycles of two seconds suction and two seconds relaxation. The mechanical properties of the skin were expressed as structural extensibility (*U*_*f*_), residual skin deformation (*U*_*f*_ − *U*_*a*_), elastic recovery (*U*_*r*_/*U*_*f*_), and immediate elasticity (*F*_0_). An increase of these parameters indicates an increase of skin extensibility and a decrease of skin recovery after stress. Cutometer measurements were performed in duplicate and in accordance to the available guidance [33].

### 2.6. Statistical Analysis

Obtained clinical scores and parameters were analysed descriptively using means and standard deviations (SDs). One-way ANOVA was performed to compare means of the three age groups. In order to identify bivariate associations between the measured skin properties, exposure variables and clinical scores, scatter plots were created. If the pattern of the dots in the scatter plots justified a possible linear relationship, Pearson's correlation coefficients were calculated. A correlation coefficient greater than 0.3 was assumed as a minimal acceptable level of bivariate association.

If bivariate correlations coefficients ranged from 0.3 to 0.8 or from −0.8 to −0.3, associations between variables were further investigated adjusted for chronological age. Regression models were built between the variables of interest with age as second predictor variable. Standardized beta coefficients of the predictor variables indicate the direction and strength of association and adjusted* R*² was used to describe the overall model fit. Only multivariable models were finally shown, if the predictor variable adjusted for age had a statistically significant influence on the dependent variable. A significance level of *P* < 0.05 was applied. All statistical analysis was performed using IBM SPSS Statistics 20.0.

## 3. Results

### 3.1. Sample Characteristics

Sample characteristics are displayed in Table 1. Mean ages of the three groups were 33.5, 55.4, and 76.6 years, respectively. All subjects had comparable body mass indices and the Fitzpatrick photo types II and III were equally distributed. In the young group no woman was postmenopausal, whereas 75% of the mid-aged and 100% of the old women were after their menopause. Mean lifetime sun exposure ranged from 48522 to 90953 hours.

### 3.2. Clinical Assessments and Biophysical Parameters

Clinical assessment scores are presented in Table 1. Means of the skin ageing scores, wrinkle scores, and sagging scores were always higher in the older groups compared to the younger groups. Differences between the age groups were statistically significant for all three scores (*P* < 0.001).

Means and SDs of the biophysical measurements are shown in Table 2. TEWL was highest in the young group and lower in the middle-aged and old-aged groups, which was statistically significant. SCH varied between 41.3 and 47.4, and skin surface pH ranged from 4.5 to 5.2 across all age groups. Differences between the groups were not statistically significant. Skin luminance (*L* ^*^) was significantly lower in the old group compared to the mid-aged and young group on both cheeks, whereas yellowness (*b* ^*^) seemed to be similar. Skin roughness increased with increasing age without statistical significance. The structural extensibility (*U*_*f*_), residual skin deformation (*U*_*f*_ − *U*_*a*_), and immediate elasticity (*F*_0_) increased with age, whereas elastic recovery (*U*_*r*_/*U*_*f*_) decreased with age.

Mean values of all biophysical measurements were highly comparable between the right and left half cheek. Also the *P* values of the ANOVA showed similar probabilities.

#### 3.2.1. Bivariate Associations

**Figure 1:**
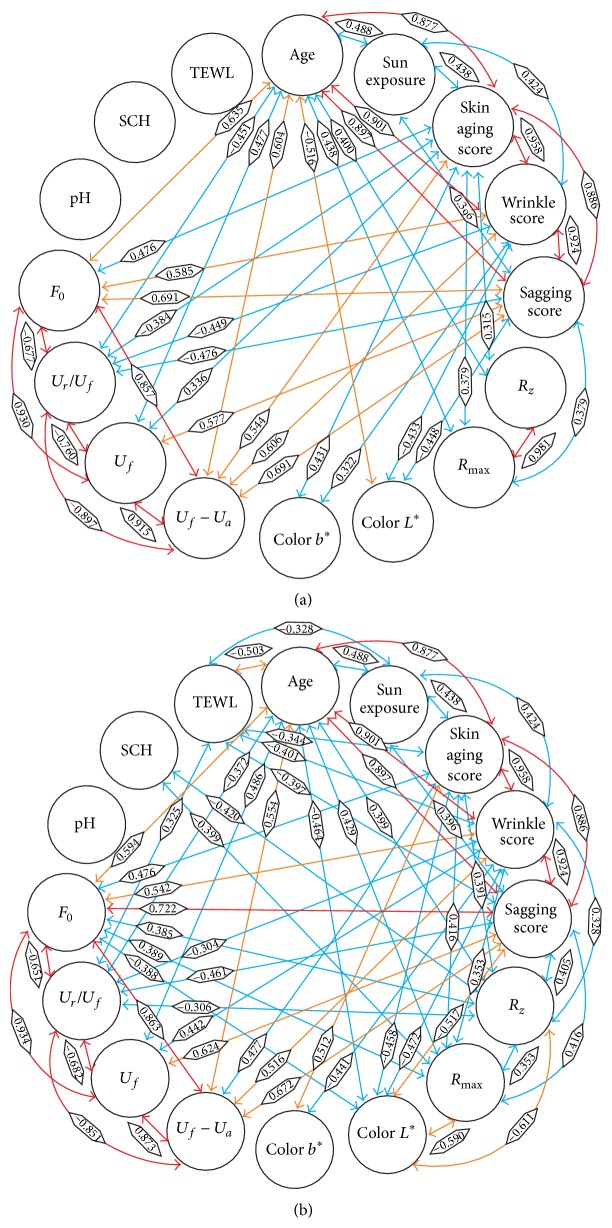
Bivariate correlations between parameters on the right (a) and left (b) cheeks. Associated parameters are connected by an arrow. Pearson's correlation coefficients are presented on the arrows. The colour of the arrow indicates the strength of association between the parameters (0.3 < *r* ≤ 0.5, blue; 0.5 < *r* ≤ 0.7, orange; *r* > 0.7, red).

Correlations between all measured parameters are displayed in Figure 1. On the left cheek there were more correlations >0.3 or <−0.3 between parameters (*n* = 59) compared to the right cheek (*n* = 42). Correlation coefficients ranged from −0.611 between *R*_*z*_ and *L* ^*^ on the left cheek to 0.958 between the skin ageing score and the wrinkle score. Strongest associations (*r* > 0.8) were detected between age and the skin ageing score, wrinkle score, and sagging score, between the clinical scores and between the skin elasticity values. Associations < 0.3 or >−0.3 were found for pH on both cheeks, and for TEWL and SCH on the right cheek. These low correlations were ignored in the next analysis step.

#### 3.2.2. Linear Regression Models

**Figure 2:**
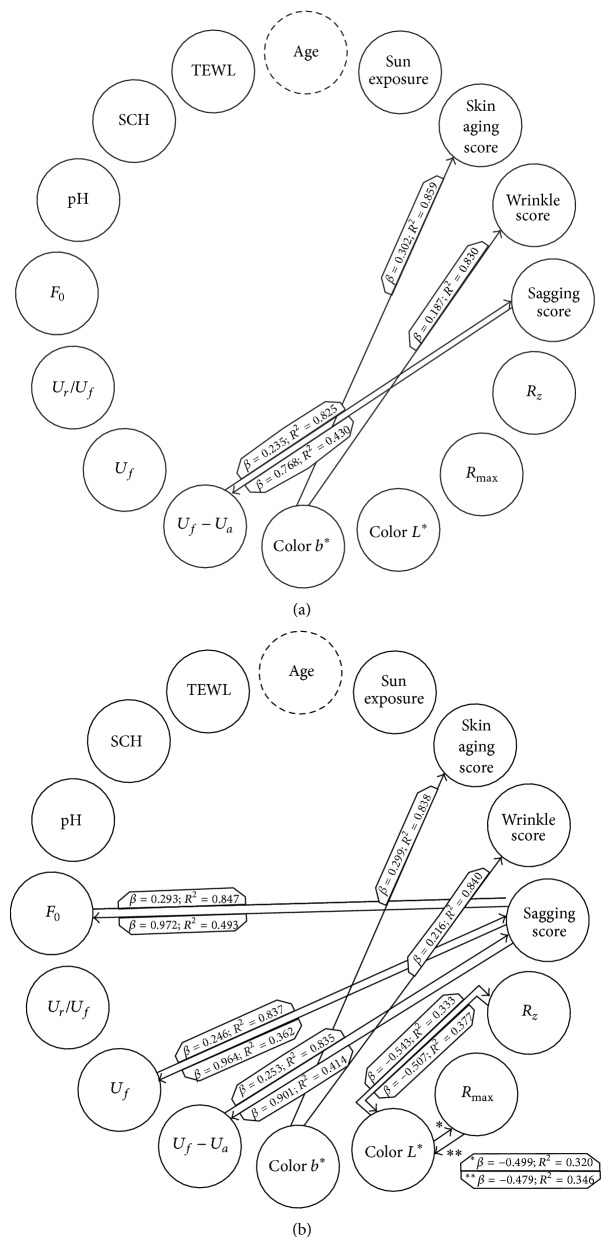
Multivariate regression models adjusted for age for the right (a) and left (b) cheeks. Statistically significant associations are presented by an arrow. Each arrow points to the dependent variable of the given model. Beta coefficients of the independent variables and the adjusted model fits (*R*²) are indicated on the arrows.

Age adjusted associations are shown in Figure 2. Twelve associations were found to be statistically significant on the left cheek and four on the right cheek. Model fits ranged from* R*² = 0.320 (*L* ^*^ and *R*_max⁡_) on the left cheek to* R*² = 0.859 (*b* ^*^ and skin ageing score) on the right cheek. Weakest associations were observed between *b* ^*^ and the wrinkle score (*β* = 0.187), and between *U*_*f*_ − *U*_*a*_ and the sagging score (*β* = 0.235) on the right cheek. Strongest associations were observed between *F*_0_ and the sagging score (*β* = 0.972) and between *U*_*f*_ and the sagging score (*β* = 0.964) on the left cheek.

## 4. Discussion

In the current study we quantified facial skin ageing in females combining the clinical and the biophysical perspective. Furthermore we tried to disentangle primarily intrinsic from extrinsic ageing phenomena.

Most of the investigated parameters were associated with chronological age which is in accordance with previous findings. For instance, we observed that lifetime sun exposure was positively correlated with chronological age. That is reasonable and in line with a previous study [34]. We observed higher skin ageing, wrinkle, and sagging scores in the aged groups, which supports the construct validity of the applied clinical scales [26–28]. We also reproduced an age-dependent increase in *R*_max⁡_ und *R*_*z*_ [35, 36]. The analysis of the skin colour revealed a significant reduction of skin luminance (*L* ^*^ value) with age, which is in concordance with previous studies that reported a diminution of this parameter with age on the face of female subjects [37, 38]. An increase in skin extensibility with age was also previously shown [18, 39], which might be explained by a loss of elastic fibres and changes in the extracellular matrix during skin ageing [39]. At the same time, aged skin showed reduced elastic recovery indicating that the skin is less able to regain its original state after deformation [37]. TEWL, SCH, and pH are regarded as the most important parameters to quantify the barrier function of the skin* in vivo*. Mean TEWL decreased with age which is supported by a recent meta-analysis [15], but the strength of this relationship was weak. There were no associations between skin surface pH and SCH indicating that the facial skin barrier function seems to changes little during facial skin ageing.

Besides correlations with chronological age associations of other parameters with each other were detected. The four measured skin elasticity parameters express associated properties of the skin and thus their correlations with each other were high. The same was true for the roughness estimates. The high interrelatedness of the three clinical scales was shown for the first time. Although conceptually distinct, the clinical phenomena facial skin ageing, wrinkling and sagging seem to be closely related to each another in the investigated sample. Interestingly, in our study lifetime sun exposure showed clear associations only with chronological age and the clinical scores on both cheeks. This indicates that UV-exposure might have a minor impact on other skin structure or barrier characteristics. This was an unexpected finding. Indeed several studies have shown that biomechanical and skin barrier parameters are different on sun exposed and sun protected skin areas [4, 15, 37, 40]. However, our findings indicate that the variable chronological age seems to have the most important impact on biophysical characteristics of facial skin than the accumulation of UV exposure.

Measured parameters and patterns of associations were similar on the left and right cheek skin. This indicates that despite possible asymmetrical sun exposures skin ageing related changes are highly comparable on both body sites. The similarity of our findings between the left and right cheek skin supports the internal validity of the study results.

In order to statistically reduce the influence of intrinsic ageing, the analyses were rerun adjusted for the variable chronological age. After that, 85 (84%) of the bivariate associations were no longer detectable. This indicates that the majority of bivariate associations could be explained by intrinsic ageing (chronological age) to a large extent. Depending on the direction of the multivariable associations beta coefficients of the remaining models were mostly lower than the bivariate associations. This again indicates the strong influence of chronological age on nearly all bivariate associations.

A major finding on both cheeks was that the degree of skin “yellowness” (*b* ^*^) predicted the skin ageing and wrinkle scores after adjustment to chronological age. This might be explained by the accumulation of collagen- and elastic fibre crosslinks caused by lifelong UV damage, which leads to the appearance of wrinkles and an overall aged appearance [9]. Another possible explanation is the thinning of epidermis which may lead to a translucency of supporting fat and to the formation of wrinkles. Similarly, associations between residual skin deformation and the clinical phenomenon of sagging remained statistically significant on both cheeks after adjustment for age. This might be caused by thinning of the epidermis and a lower amount of elastic fibres in the dermis [41], which results in changes in skin stiffness properties. Skin thickness and dermal collagen content is also decreased in postmenopausal women due to hypoestrogenia [42, 43]. Thus decreased elastic recovery may also be an effect of hormonal changes during menopause, in which all women of the oldest group of our study had been.

Our finding indicates, that residual skin deformation may be predicted by clinical evaluation without instrumental measurement and vice versa. A surprising finding of this study was the negative association between skin roughness and luminance, which persisted after adjustment for age on the left cheek. Probably smoother skin appears brighter because more light is being reflected. Taken together, only skin yellowness, the clinical skin ageing scores, and skin elasticity seem to be “really” related to each other independently from chronological age. Thus these parameters seem to be mainly influenced by extrinsic factors.

### 4.1. Limitations

This study was conducted in a small number of subjects to evaluate possible bi- and multivariate associations between different skin parameters. However, a larger study is needed to confirm results. Our study provides the evidence for effect sizes that are needed for future sample size calculations. We included female subjects only in order to reduce possible gender bias. Whether findings are applicable to males is unclear. Similarly our findings might be applicable for light skin tones only. The strength of correlations might have been influenced by the ranges of the applied scores and measurements. For instance a slight increase of the sagging score (range 0 to 4) had a stronger influence on residual deformation expressed in millimeters than vice versa.

## 5. Conclusion

This is the first study showing associations between multiple biophysical and clinical parameters of the facial skin with chronological age and lifetime sun exposure in a sample of different age groups. We demonstrated strong associations of chronological age with most parameters. However, after statistical adjustment for chronological age only few associations remained. This indicates that the variable chronological age as a surrogate for intrinsic ageing has a very strong influence on facial skin characteristics in Caucasian women in general. Relationships between skin colour, clinical scores, and skin elasticity seem to exist independently from chronological ageing and thus seem to be mainly influenced by extrinsic factors.

## Figures and Tables

**Table 1:** Sample characteristics and clinical assessment scores.

	Young group (*n* = 8)	Midaged group (*n* = 8)	Old group (*n* = 8)	Total (*n* = 24)	ANOVA *P* value
Age in years,					
Mean (SD)	33.5 (2.1)	55.4 (2.7)	76.6 (1.9)	55.2 (18.1)	n.a.
Range	31 to 37	51 to 59	74 to 79	31 to 79	
BMI in kg/m^2^,					
Mean (SD)	21.8 (2.0)	26.0 (5.0)	26.2 (2.5)	24.7 (3.9)	n.a.
Range	19.7 to 25.9	20.8 to 30.5	21.1 to 29.3	19.7 to 30.5	
Photo type, *n*					n.a.
II	4	7	4	14	n.a.
III	4	1	4	10	n.a.
Postmenopausal, *n*	0	6	8	14	n.a.
Lifetime sun exposure in hours, mean (SD)	48522 (17039)	62141 (11934)	90953 (47621)	67205 (33888)	n.a.
Skin ageing score, mean (SD)	19.6 (1.6)	25.7 (3.8)	32.5 (3.9)	—	<0.001
Wrinkle score, mean (SD)	26.8 (7.9)	56.4 (12.3)	83.3 (14.6)	—	<0.001
Sagging score,					
Mean (SD)	1.3 (0.4)	2.4 (0.4)	3.6 (0.6)	—	<0.001
Median	1.5	2.5	4.0		

SD: standard deviation; BMI: Body Mass Index; n.a.: not applied.

**Table 2:** Means and standard deviations of biophysical measurements and the difference between the age groups (ANOVA) for the right and left cheeks.

	Young group (*n* = 8)		Midaged group (*n* = 8)		Old group (*n* = 8)		ANOVA *P* value	
	Right	Left	Right	Left	Right	Left	Right	Left
Skin barrier								
TEWL	12.9 (3.6)	14.2 (3.5)	8.9 (1.8)	9.0 (2.0)	9.8 (2.7)	9.8 (2.4)	0.023	0.002
SCH	41.3 (11.1)	45.6 (12.1)	47.4 (9.4)	46.0 (10.7)	42.2 (10.7)	42.1 (12.0)	0.463	0.760
pH	4.9 (0.6)	4.5 (0.7)	5.2 (0.5)	4.9 (0.6)	4.8 (0.4)	4.8 (0.3)	0.236	0.323
Skin colour								
*L* ^*^	65.8 (1.4)	65.2 (1.8)	64.6 (2.4)	64.9 (1.8)	62.2 (3.3)	62.9 (2.0)	0.028	0.042
*b* ^*^	15.8 (1.3)	15.9 (1.3)	17.7 (2.1)	17.8 (1.9)	16.3 (1.8)	17.0 (2.1)	0.101	0.118
Skin roughness								
*R*_*z*_	47.5 (7.3)	47.4 (5.6)	48.9 (5.4)	49.6 (9.5)	54.6 (8.8)	54.3 (6.1)	0.140	0.175
*R*_max⁡_	63.4 (9.4)	63.7 (7.0)	64.4 (7.9)	67.3 (12.4)	73.7 (10.5)	73.3 (7.8)	0.076	0.141
Skin elasticity								
*U*_*f*_	0.21 (0.04)	0.21 (0.04)	0.26 (0.09)	0.25 (0.08)	0.31 (0.09)	0.29 (0.07)	0.071	0.074
*U*_*f*_ − *U*_*a*_	0.06 (0.01)	0.07 (0.02)	0.1 (0.04)	0.08 (0.03)	0.13 (0.06)	0.11 (0.04)	0.011	0.035
*U*_*r*_/*U*_*f*_	0.29 (0.02)	0.28 (0.04)	0.24 (0.04)	0.26 (0.03)	0.22 (0.09)	0.25 (0.05)	0.082	0.246
*F*_0_	0.04 (0.01)	0.04 (0.01)	0.05 (0.01)	0.05 (0.01)	0.06 (0.01)	0.06 (0.01)	0.004	0.011

TEWL: transepidermal water loss (g/m²/h); SCH: stratum corneum hydration (arbitrary units); *L* ^*^: luminance; *b* ^*^: yellowness; *R*_*z*_: mean depth of roughness (*µ*m); *R*_max⁡_: maximal roughness (*µ*m); *U*_*f*_: structural extensibility (mm); *U*_*f*_ − *U*_*a*_: residual skin deformation (mm); *U*_*r*_/*U*_*f*_: elastic recovery (ratio); *F*_0_: immediate elasticity (area).
